# X-ray Induced Fragmentation of Protonated Cystine

**DOI:** 10.1021/acs.jpca.1c10158

**Published:** 2022-02-25

**Authors:** Geethanjali Gopakumar, Pamela H. W. Svensson, Oscar Grånäs, Barbara Brena, Lucas Schwob, Isaak Unger, Clara-Magdalena Saak, Martin Timm, Christine Bülow, Markus Kubin, Vicente Zamudio-Bayer, J. Tobias Lau, Bernd von Issendorff, Abdul R. Abid, Andreas Lindblad, Emma Danielsson, Ebba Koerfer, Carl Caleman, Olle Björneholm, Rebecka Lindblad

**Affiliations:** † Department of Physics and Astronomy, Uppsala University, Box 516, SE-751 20 Uppsala, Sweden; ‡ Deutsches Elektronen-Synchrotron DESY, Notkestrasse 85, DE-22607 Hamburg, Germany; ¶ Department of Physical Chemistry, University of Vienna, Währingerstraßze 42, 1090 Vienna, Austria; § Abteilung für Hochempfindliche Röntgenspektroskopie, Helmholtz-Zentrum Berlin für Materialien und Energie, Albert-Einstein-Strasse 15, DE-12489 Berlin, Germany; ∥ Institut für Optik und Atomare Physik, Technische Universität Berlin, Hardenbergstrasse 36, DE-10623 Berlin, Germany; ⊥ Physikalisches Institut, Albert-Ludwigs-Universität Freiburg, Hermann-Herder-Strasse 3, DE-79104 Freiburg, Germany; # Nano and Molecular Systems Research Unit, Faculty of Science, University of Oulu, P.O. Box 3000, 90570 Oulu, Finland; ○ Center for Free-Electron Laser Science, Deutsches Elektronen-Synchrotron DESY, Notkestrasse 85, DE-22607 Hamburg, Germany; △ Department of Physics, Lund University, Box 118, SE-22100 Lund, Sweden; ∇ Department of Chemistry - Ångström Laboratory, Uppsala University, Box 538, SE-75121 Uppsala, Sweden

## Abstract

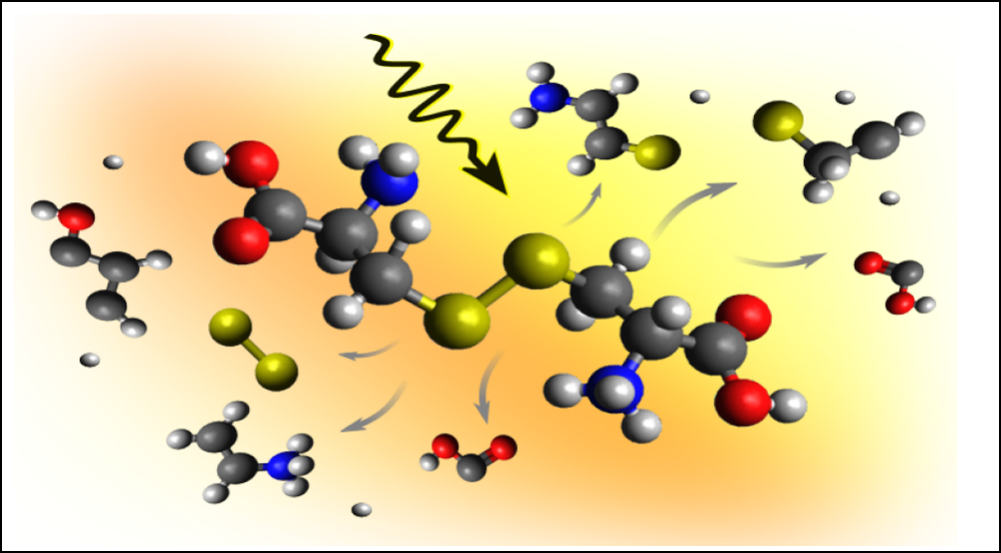

We demonstrate site-specific X-ray induced fragmentation across the sulfur L-edge of protonated cystine, the dimer of the amino acid cysteine. Ion yield NEXAFS were performed in the gas phase using electrospray ionization (ESI) in combination with an ion trap. The interpretation of the sulfur L-edge NEXAFS spectrum is supported by Restricted Open-Shell Configuration Interaction (ROCIS) calculations. The fragmentation pathway of triply charged cystine ions was modeled by Molecular Dynamics (MD) simulations. We have deduced a possible pathway of fragmentation upon excitation and ionization of S 2p electrons. The disulfide bridge breaks for resonant excitation at lower photon energies but remains intact upon higher energy resonant excitation and upon ionization of S 2p. The larger fragments initially formed subsequently break into smaller fragments.

## Introduction

**Figure 1:**
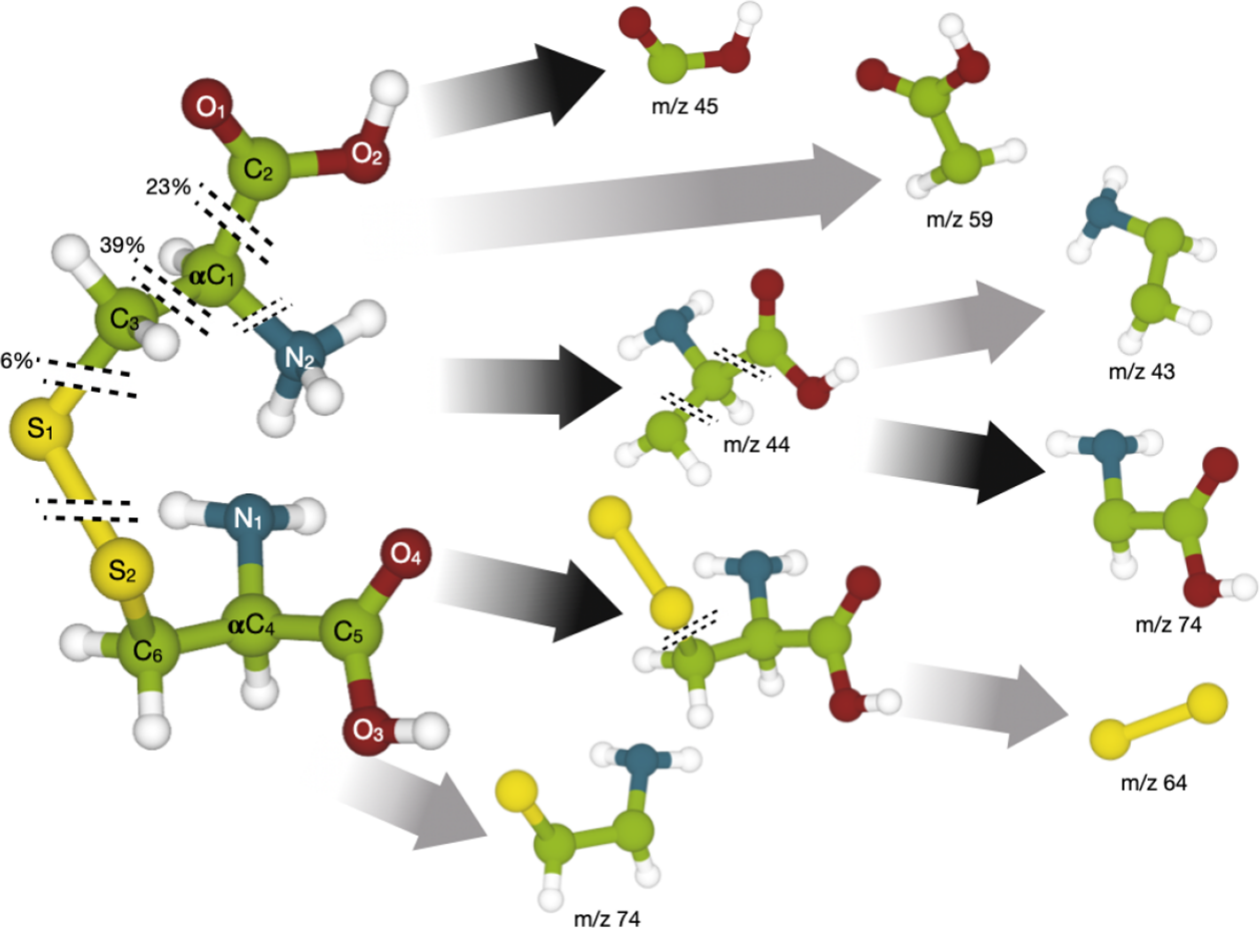
Structure of the cystine molecule and the most relevant fragmentation pathways. The percentage marks probabilities for the breaking of bonds as estimated from the molecular dynamics simulations above the ionization threshold (173 eV), and their corresponding fragments are indicated with black arrows. Fragments with assigned masses are observed in the experimental mass spectrum.

The interaction of radiation with biomolecules in the gas phase has gained attention in recent years^1^ since the radiation-induced electronic and nuclear dynamics often lead to fragmentation of the molecule. The microscopic picture of such dynamics of radiation damages in biomolecules is a pivotal piece of information in the field of biology, biotechnology, astrochemistry, and astrobiology.^2−5^ Proteins are biomolecules that drive many of the biochemical processes of life, and the functioning of a protein is governed by its structure. Protein molecules are large polymers built up by amino acids, and deciphering the radiation-induced electron dynamics in a protein molecule with a complicated structure is quite challenging. A “bottom-up” approach is to study the dynamics in a smaller part of the protein, such as an amino acid or di-, tri-, or oligopeptides. One structurally interesting part of proteins is sulfur bridges. Those are important for structural stability and are among the stronger intermolecular bonds in a protein. From a radiation point of view, they are different from the rest of the molecules, due to the fact that the ionization cross-section for sulfur is different from those for carbon, oxygen, and nitrogen. Measurements of photoionization and fragmentation as well as NEXAFS (Near Edge X-ray Absorption Fine Structure) of amino acids have been reported in the literature,^6−11^ but studies of the sulfur bridge containing molecules are, to the best of our knowledge, rare. In this study, we focus on photon-induced fragmentation of two cysteine molecules linked together with a sulfur bridge, creating a cystine molecule. Here we measure the ion yield NEXAFS at the sulfur L-edge of protonated cystine in the gas phase—see the structure in Figure 1—and compare it to simulations of the same system. By doing so, we can provide new insights into the fragmentation dynamics initiated by the ionization.

## Methods

### Experimental Section

Commercially purchased cystine (from Sigma-Aldrich, purity >99%) was used for the study. The molecules were dissolved in 50:50 mixtures of water:acetonitrile. Then, 50 μL of formic acid was added for protonation. The experiments were carried out at the Ion Trap endstation at the UE52-PGM beamline of the BESSY II synchrotron radiation facility operated by the Helmholtz-Zentrum Berlin.^12,13^ The protonated molecules were transferred from the solution into the vacuum ion trap set up using electrospray ionization (ESI).^14^ The cationic species were first directed to a quadrupole mass filter, where the desired ion mass-to-charge ratio was selected. The selection of protonated cystine was confirmed by the *m*/*z* 241.3 peak in the mass spectrum. The selected ions were guided to the radiofrequency (RF) linear ion trap, where they were accumulated and exposed to linearly polarized X-rays. The trap content was extracted in short bunches to a reflectron time-of-flight mass spectrometer with a nominal resolution of 3000 Da.^12^ The trap was cooled to 15 K, and the ions were thermalized in a helium buffer gas. The residence time in the trap was in the order of a few seconds.

After X-ray absorption, the core excited state primarily relaxes via Auger decay followed by fragmentation of the molecular cations. The fragment mass spectra were calibrated using the *m*/*z* 64 and *m*/*z* 45 peaks. The fragmentation products were used to record action spectra in partial ion yield while scanning the photon energy across the sulfur L-edge. The photon energy was scanned in steps of 40–70 meV. A beamline exit slit of around 200 μm was used, giving estimated bandwidths of approximately 50 meV for the sulfur L-edge. The partial ion yield spectra were summed and the resulting spectrum is comparable to the NEXAFS spectrum. Possible contribution to the NEXAFS spectrum resulting from X-ray absorption of the fragments is estimated to be less than 1%.^12^ Even though sequential valence photoionization has been observed in the VUV photon energy range when studying large clusters using this ion trap setup,^15^ it has not been observed for core level photoionization in the soft X-ray range.^16,17^

### Computational Methods

We have performed simulations of the photon-absorption process as captured by the NEXAFS spectrum using the ORCA^18^ code. We used the framework of density-functional theory based on restricted open-shell configuration interaction singles (DFT-ROCIS). The sulfur 2p states are assumed to be initial states, and all possible excited states combinations are taken into account within the CI single approximation. Spin–orbit splitting is included to account for the L_2_/L_3_ splitting. A basis set of def2-TZVP^19^ was used, and the open-shell spin restricted Kohn–Sham equations were solved with the B3LYP-functional.^20^

Density Functional Theory (DFT) was adopted for geometry relaxation and orbital visualization to visualize the molecular orbitals. The calculations were performed using Gaussian 16 quantum chemistry software^21^ with B3LYP exchange-correlation functional in combination with 6-31G(d,p)^22^ basis set. Unoccupied molecular orbitals were computed in the molecular ground state. Above the ionization threshold, we have employed Born–Oppenheimer Molecular Dynamics simulations of the cystine molecule at a charge state of +3. First, we generate thermalized configurations of cystine using classical molecular dynamics of the positively charged cystine molecule with one extra proton at one of the nitrogen sites. These simulations were done using the molecular dynamics tool GROMACS,^23^ using the Generalized Amber Force Fields (GAFF).^24^ All interaction parameters were created the same way as described by Caleman et al.^25^ A 1 ns simulation in vacuum was performed from which 20 configurations were saved and used as starting structures for the Born–Oppenheimer Molecular Dynamics simulations. In short, we did a presimulation with a Berendsen thermostat^26^ set to 300 K and a coupling parameter to 0.1 ps. From the presimulation, we took the final structure and velocities and used them as our starting point in the production run, where no temperature coupling was used. From the 1 ns production run, we dropped frames that were at least 1 ps apart and used them as individual starting structures for the Born–Oppenheimer simulations.

The fragmentation process was simulated using Born–Oppenheimer molecular dynamics simulations as implemented in the *Siesta*-package.^27^ We simulate 18 different starting conditions for one picosecond, resulting in some variability in the final outcome. The level of ionization is assumed to be +3, resulting from the initial photoionization and subsequent Auger process. The electronic levels are occupied according to the Aufbau principle, on the level of density-functional theory. We use the exchange-correlation functional by Perdew, Burke, and Ernzerhof (PBE).^28^ The core electrons and corresponding modifications of the valence wave functions are described using norm-conserving pseudopotentials of the Kleinman–Baylander type.^29^ The molecular orbitals of the ionized molecule are described with a double-zeta + polarization orbitals basis set and a grid cutoff of 70 Ry for numerical integration. The time-step of the Verlet time-stepping algorithm is set to 0.5 fs to adequately capture the rapid movement of hydrogen atoms.

The main analysis done on the fragmentation simulations trajectories was to investigate what bonds in the molecule broke during the simulations. We use the so-called Souvatzis integrity parameter , which previously has been used to define if a bond is considered broken or not.^30^ Here we have developed the expression for , such that it captures anharmonic contributions to the bond length. Instead of analyzing the deviation from the distance between two atoms in a relaxed ground state configuration at the initial step of the simulation, we define to use the equilibrium bond length acquired from the thermalization simulations. That is, the bond distance between atoms [A,B] at time zero, *d*_*i*_[A,B](0), was replaced with the mean value μ(*d*_*i*_^Therm.^[A,B]) from the thermalization runs shifted by one standard deviation σ(*d*_*i*_[A,B]). The shift was done because the function is not symmetric around the mean value. Using the mean value of bond distances surpasses the use of bond distance at time zero because the bonds fluctuate and could therefore return significantly varying values for a specific time. The following formula was used
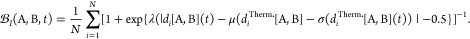
1

The parameter is 0 for a broken bond and 1 for an intact bond.

## Results and Discussion

**Figure 2:**
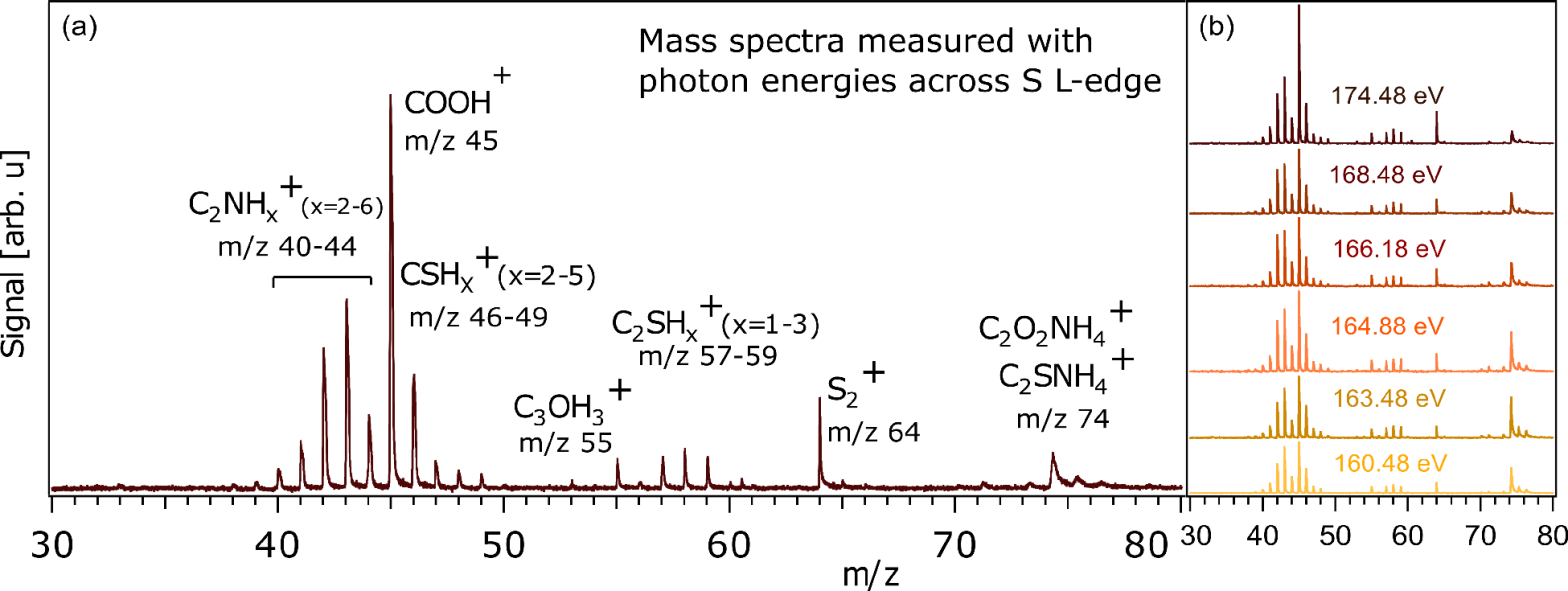
(a) Fragment mass spectrum measured with an incident photon energy of 174.48 eV at the sulfur L-edge. Peak assignments are discussed in the text. (b) Mass spectra measured at various photon energies across the S L-edge.

The mass spectra of protonated cystine (*m*/*z* 241.3) were measured using photon energies (*h*ν) of 160–178 eV across the sulfur L-edge. The lower photon energies resonantly excite an S 2p electron to bound excited state orbitals. The core-excited state subsequently decays via Auger decay within a few femtoseconds into a + 2 state with two valence holes, leading to fragmentation of the molecule. On further increasing the photon energy, the molecules are instead ionized directly, followed by Auger decay, which increases the charge on the molecule further to +3 from the initial protonated +1 state. The transition from exciting to bound excited states vs direct ionization results in slightly different fragmentation processes. The fragmented cationic species observed in the mass spectra constitute the peaks of mass/charge (*m*/*z*) ratio as shown in Figure 2a. The mass window was chosen to show as many fragment peaks as possible with good resolution.

The most intense peak shows a fragment at *m*/*z* 45, which we assigned to a singly charged carboxyl group (COOH^+^). Fragmentation studies of other neutral amino acids, like proline and alanine^31,32^ and the monomer cysteine,^11^ have also shown the formation of COOH^+^. The fragment peaks at *m*/*z* 40–44 are assigned to C_2_NH_*x*_^+^ (*x* = 2–6) originating from breaking of C1–C2 or C4–C5 and C3–S1 and C6–S2 bonds (see Figure 1 for atom notation) in conjunction with severing the carboxyl group. These fragments were also observed in the radiation-induced fragmentation of gas-phase alanine.^32^ The fragment at *m*/*z* 55 is assigned to C_3_OH_3_^+^ and the *m*/*z* 64 peak is attributed to a singly charged disulfide bridge (SS^+^) broken off intact from the dimer. We note that none of these fragments contains a single sulfur atom and they can therefore be formed without breaking the disulfide bridge. Single sulfur atoms are, on the other hand, found in the C_2_SH_*x*_^+^ (*x* = 1–3) fragments that form the peaks at *m*/*z* 57–59 and the CSH_*x*_^+^ (*x* = 2–5) fragments forming the *m*/*z* 46–49 peaks. The fragment peak at *m*/*z* 74 requires a more thorough discussion. A collision-induced fragmentation study of protonated cystine using high-resolution mass spectrometry has shown that the *m*/*z* 74 peak is composed of two isobaric fragments, C_2_SNH_4_^+^ (*m*/*z* 74.00590) and C_2_O_2_NH_4_^+^ (*m*/*z* 74.02365).^33^ As will be discussed further below, we see indications that resonant excitations lead to the formation of the C_2_SNH_4_^+^ fragment, while direct ionization mostly leads to the formation of the C_2_O_2_NH_4_^+^ fragment.

In Figure 2b, we show the mass spectra obtained at different photon energies around the sulfur L-edge and we see a clear variation in peak intensity. The *m*/*z* 74 peak intensity decreases while the *m*/*z* 45 peak increases with increasing photon energy, indicative of a competition between the two fragments depending on if the sulfur 2p electron is resonantly excited to higher molecular orbital or ionized. In a similar fragmentation study on the monomer cysteine,^11^ on ionizing at the carbon, nitrogen or oxygen K-edge the *m*/*z* 45 COOH^+^ fragments were formed most abundantly, while at the S L-edge ionization other dissociation channels have similar intensity. From Figure 2b, it can also be seen that the formation of disulfide fragments at *m*/*z* 64 was strongly increased at higher photon energies.

**Figure 3:**
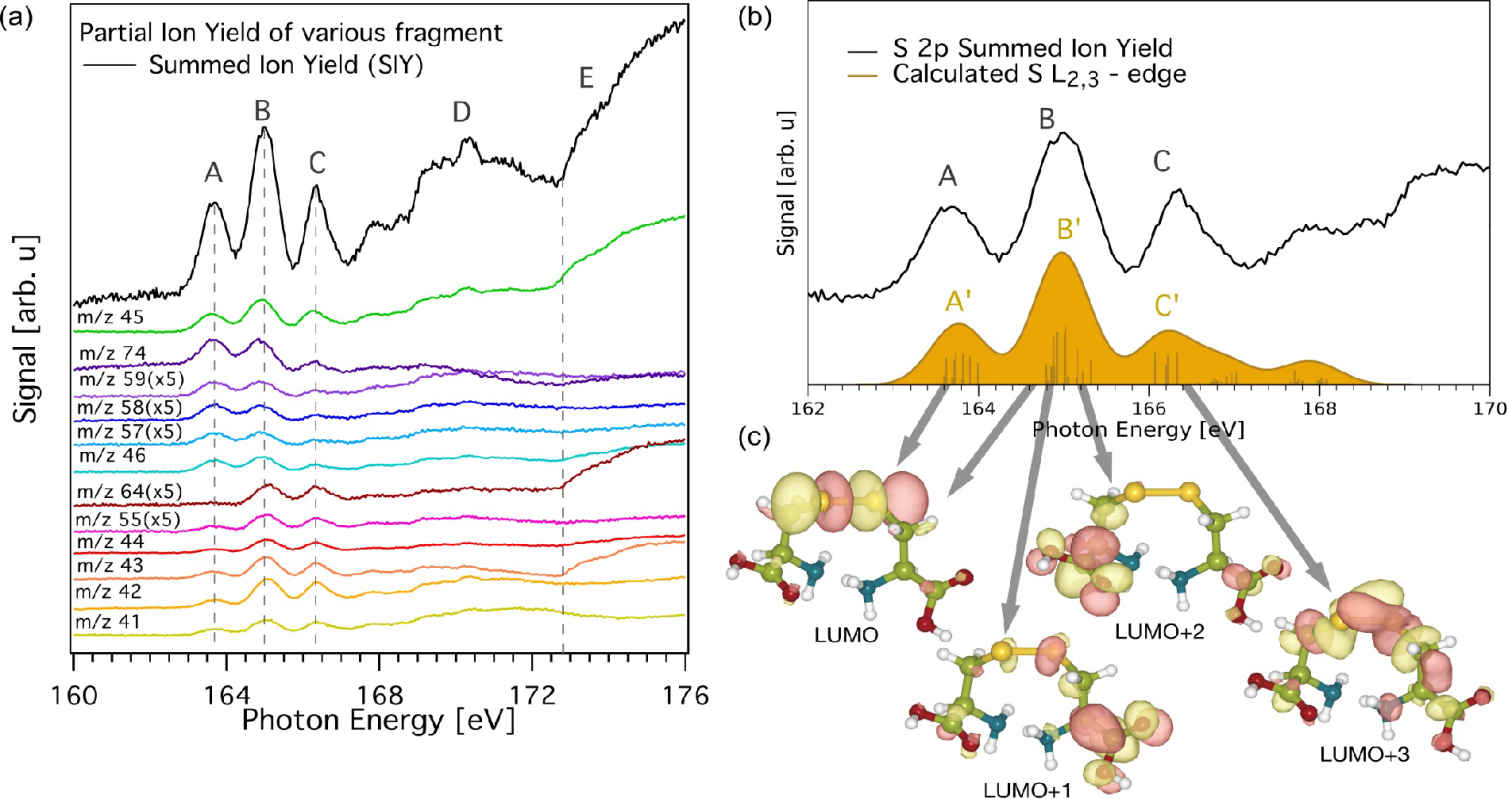
(a) Summed and partial ion yield of cystine on excitation at the sulfur L-edge. The partial ion yields are scaled to distinguish the different peaks it respectively contributes to and are grouped according to their most intense resonant excitation. (See Supporting Information for unscaled spectra.) The different excitations are labeled and the details are discussed in the text. (b) Comparison between SIY and calculated S L_2,3_-edge with each electronic transition represented as bars. For a better comparison with the experimental broadening, the NEXAFS spectrum was obtained by convolution of the bar graph using a Gaussian function of 0.7 eV full width at half-maximum, and shifted by 7.23 eV to match the experimental SIY. (c) Calculated LUMO ground state levels for cystine and the corresponding visualization of the LUMO, LUMO+1, LUMO+2, and LUMO+3. For the illustration, an isovalue of 0.04 e/Å^3^ was used. The figure illustrates that the DFT calculations of the LUMO levels reproduce the main features of the experimental total ion yields, and how the orbitals differ between the four LUMO levels.

A more detailed view of the photon energy dependence is found in the partial ion yield spectrum (PIY), obtained as the integrated intensity of one fragment peak as a function of photon energy. PIY of sulfur L-edge spectra for the most intense fragments is found in Figure 3a. By summing the PIY for all fragments observed, shown in Figure 2, the summed ion yield (SIY) spectrum was produced; see Figure 3, parts a and b. As possible smaller, or neutral, fragments were not taken into account, the SIY is not exactly the same as the total ion yield. Also, more than one fragment might be formed in one absorption event, yet the SIY is a good approximation of the NEXAFS spectrum. As seen in parts a and b of Figure 3, the S L-edge spectra consist of three peaks around 164–168 eV (labeled A, B, and C respectively), a broad feature around 168–173 eV (labeled D) and a steep rise starting at 173 eV (labeled E). In molecules containing a single S atom, the three distinct peaks A, B, and C have been assigned to electronic transitions from the spin–orbit split S 2p core level to unoccupied molecular orbitals involving C–S bonds.^34,35^ The relative intensity of the second peak B is higher compared to A and C as it is the overlap of 2p_1/2_ and 2p_3/2_ to different unoccupied molecular orbitals. The broad feature D is also resonant excitation to unoccupied molecular orbitals, as can be seen in the calculated NEXAFS spectrum, as well as found in the literature.^35^ The onset at 173 eV of the high-intensity feature E is the ionization threshold for S 2p ionization in cystine. This is in reasonable agreement with the ionization energy of other small sulfur-containing molecules, which has a value of 171 eV.^36^

To investigate the final state of the excited S 2p-electron, we simulate the NEXAFS spectrum of the relaxed protonated cysteine molecule. As seen in Figure 3b, the agreement between the simulated spectrum and the SIY below the ionization threshold is very good. The simulated NEXAFS spectrum is composed of all possible many-body excitations within an active space formed of the S 2p electrons and all unoccupied single electron orbitals. The peak labeled A′ in the spectrum consists of excitations mainly involving S 2p_3/2_-states excited to the lowest unoccupied molecular orbital (LUMO) orbital, which has an antibonding S–S character. The B′ peak contains the contribution of 2p_3/2_ states excited into predominantly LUMO as well as 2p_1/2_ excited to a collection of orbitals involving LUMO+1, LUMO+2, and LUMO+3. In comparison to the LUMO, these orbitals have more weight around the S–C bond. Peak C′ involves 2p_3/2_ excitations to the aforementioned orbitals as well as higher-lying orbitals. The measured spectrum shows a stronger increase than the calculated one at energies in the range 168 eV to the ionization threshold. This increase we associate with the increasing importance of Rydberg states.

The LUMO, as well as the three sequential levels, LUMO+1, LUMO+2, and LUMO+3, are depicted in Figure 3c. In contrast to the methionine and thioxanthene derivatives presented in previous studies,^34,35^ cystine contains a sulfur bridge. Here, the LUMO orbital has a dominant S–S contribution and locally resembles an antibonding σ* combination of S 3p orbitals, which can be expected to be dissociative along with the S–S bond. This S–S contribution is small in LUMO+1 and LUMO+2. According to previous experimental-theoretical studies on small molecules like thiophene, benzothiophene and dibenzothiophene, the S L-edges resonances at energies up to about 165 eV can be attributed to the electronic transition to the LUMO to LUMO+3 molecular orbitals.^37^

The PIY spectra shown in Figure 3a indicate the contribution of each fragment to the SIY. The PIY of the *m*/*z* 45 COOH^+^ fragment mimics the SIY in the given energy window, which shows the importance of this fragmentation pathway. The fragments with *m*/*z* 74, 59, 58, 57, 48, and 46 show a relatively higher intensity at peaks A and B compared to the third peak C, Figure 3a. We note that these fragments can be formed after breaking the sulfide bridge. From the simulated NEXAFS spectra, we have already seen that LUMO is along the sulfur bridge with antibonding σ* character. This can be interpreted as a correlation between resonant excitation to molecular orbitals of S character and a breaking of the sulfide bridge. Although the *m*/*z* 74 fragment was seen to be constituted by isobaric C_2_SNH_4_^+^ and C_2_O_2_NH_4_^+^ fragments in collision-induced studies,^33^ in our results, this fragment is formed mostly by the resonant excitation to LUMO, resulting in the breaking of the disulfide bond. Formation of the C_2_O_2_NH_4_^+^ fragment would require S–C bond breaking, resulting in, e.g., the SS^+^ fragment, which should then contribute to the first resonant peak A in Figure 3a. This is not the case here and therefore, we identify the *m*/*z* 74 fragment to be C_2_SNH_4_^+^ when formed in conjunction with resonant excitations below the ionization threshold. We envisage the process as stretching of the sulfur bridge during the few femtosecond lifetimes of the locally antibonding state formed upon S 2p to LUMO excitation, making the sulfur bridge prone to break after Auger decay into +2 states.

The PIY of the *m*/*z* 64, 55, and 40–44 fragments have a relatively higher intensity of resonance peak B and C, compared to peak A, Figure 3a. The *m*/*z* 64 fragment is formed by the rupture of both S–C bonds, resulting in an intact disulfide (SS^+^) bridge. This is explained by the final state of the resonance peak A, involving the occupation of the LUMO with antibonding S–S character, thus rupturing the disulfide bridge, whereas the higher resonances also involve final states that are of a different character. After removing the SS^+^ fragment, the residual fragments should be forming a peak at *m*/*z* 88, which we do not observe. It is therefore likely that this fragment undergoes further dissociation. The amine group and hydroxyl group breaking would give the *m*/*z* 55 (C_3_OH_3_^+^) fragment. The breaking of carboxyl group instead, forms the fragments at *m*/*z* 40–44 (C_2_NH_*x*_^+^ (*x* = 2–6)). The MO calculations show that the LUMO+1, LUMO+2, and LUMO+3 have higher weight around the S1–C3 (S2–C6) and C1–C2 (C4–C5) bonds. It appears that electronic excitation to these orbitals results in rupture of S–C and C–C bonds creating fragments where the sulfide bridge is allowed to stay intact. We thus see that the resonant excitations into specific orbitals with different characters lead to a degree of propensity in the fragmentation, as exemplified by the sulfur bridge breaking upon excitation into the local antibonding LUMO orbital. A fragmentation study on a similar molecule, dimethyl disulfide (CH_3_SSCH_3_), on S 2p ionization showed similar results, where the first resonant excitation of S 2p electrons to a σ*SS state produces fragments from breaking of the S–S bond while S_2_^+^ fragments are observed with excitation to higher molecular orbitals.^38^

We now turn to the photon energy above the ionization threshold labeled E in Figure 3a, which via Auger decay rapidly leads to a multitude of +3 valence hole states. From Figure 3a, we observe that it is mainly the *m*/*z* 74, 64, 46, 45, and 43 fragments that contribute to resonance E. The presence of the *m*/*z* 64 (SS^+^), *m*/*z* 45 (COOH^+^), and *m*/*z* 43 (C_2_NH_5_^+^) fragments indicate that the fragmentation pathway after core-ionizing the S 2p level results in breaking the S1–C3 (S2–C6) and C1–C2 (C4–C5) bonds. As will be discussed below, this is also the fragmentation pathway provided by the MD simulations. If ionization predominantly breaks S–C and C–C bonds, the *m*/*z* 74 fragments should in this case be associated with the C_2_O_2_NH_4_^+^ fragment, isobaric with the C_2_SNH_4_^+^ fragment discussed above.^33^ We speculate that the *m*/*z* 74 fragment peak has a different origin depending on the photon energy and that the C_2_SNH_4_^+^ fragment is formed upon resonant excitation to LUMO, populating antibonding states, while the C_2_O_2_NH_4_^+^ fragment is formed upon ionization and depopulation of bonding states. This implies that photon energies above the S 2p ionization threshold might not break the disulfide bridge. However, a small intensity increase of the *m*/*z* 46 (CSH_2_^+^) fragment above 173 eV could indicate that some breaking of the disulfide bridge does occur above the ionization threshold. Since the contribution from other fragments with broken disulfide bridge is quite small at these energies, the major fragmentation likely happens by the S1–C3 (S2–C6) and C1–C2 (C4–C5) bond breakage.

We study the time-resolved dynamics of cystine fragmentation above the ionization threshold at 173 eV with Born–Oppenheimer Molecular Dynamics. Here we assume that there is no bound excited states present, and simulate the fragmentation process assuming that the electronic system is relaxed into a high-temperature ionized state corresponding to a total charge of 3+. These assumptions are valid if the fragmentation process is slow on the time scale of the electronic relaxation process. Concerning the experiment, this corresponds to the state of protonated cystine after the ionization of S 2p orbital and emission of one Auger electron in the core-relaxation process. In Table 1, we list the percentage of simulations where a specific bond is broken, based on the bond integrity parameter, , as defined in the eq 1. The statistics in Table 1 are based on 18 simulations starting at different initial conditions, represented by ionic positions and velocities drawn from a thermalized MD trajectory of the protonated cystine molecule (see Computational Methods). Our fragmentation simulations have limitations in terms of statistical sampling and the time scales that we can simulate and the simulations are 1000 fs. The experimental observations are done on the order of ms after the initial photon but the presence of He buffer gas is likely to quench slow fragmentation channels.^39^ Within our simulated time scale, we observe three types of bond breaking:

1. The most common bond to break is the one between the carbon connected to the amine group and the carbon connected to the sulfur (C4–C6 and C1–C3). Breaking this bond leads to the production of a C_2_O_2_NH_4_^+^ fragment. This fragment has an *m*/*z* of 74, corroborating our speculation that the *m*/*z* 74 fragment likely has a contribution from C_2_O_2_NH_4_^+^ above the ionization threshold. The fragment may fall apart even further, which would lead to the production of COOH^+^ at *m*/*z* 45 and CNH_3_ (which is neutral and not captured by the experiment).
2. The second most common bond breaking that occurs in the simulations is the formation of the carboxyl group fragments, COOH^+^ (by breaking the bonds C1–C2 and C3–C4). In the experimental mass spectrum, we can see that the COOH^+^ peak at *m*/*z* 45 is the most intense. The high intensity could indicate that COOH^+^ also is created in secondary fragmentation steps, such as the splitting of the C_2_O_2_NH_4_^+^ fragment, which is something our simulations can not tell us.
3. The simulations also predict the breaking of the bond between sulfur and the neighboring carbon (C3–S1 and C6–S2). This creates a C_3_NO_2_H_6_^+2^ fragment. If the carboxylic group is split off, we end up with C_2_NH_*x*_^+^ with *m*/*z* 40–44, which is observed experimentally. Alternatively, if the amine group is split off we end up with the fragment C_2_H_3_COOH^+^, with *m*/*z* 72, which is not observed in the experimental mass spectrum. A simultaneous breaking of the C2–C1 (or C4–C5) results in COOH^+^ and neutral C_2_H_3_.

**Table 1:** Table of Relevant Bonds, Their Probability of Breaking within 1 ps of the Simulations, and the Fragments Relevant to the Breaking of a Particular Bond^a^

bond	probability of breaking (%)	direct creation of fragments	relevant for fragments
C4–C6 and C1–C3	39	C_2_O_2_NH_4_^+^	
C1–C2 and C4–C5	23	COOH^+^	C_2_NH_*x*_^+^
S1–C3 and S2–C6	6		SS^+^

a “Direct creation of fragments” refers to the fragments that were seen in the 1 ps simulation. Only bond breaking that was detected in the simulations is listed.

The simulated fragmentation pathway above the ionization threshold thus indicates that the disulfide bridge is left intact, in agreement with the experimental observations. In addition, the bonds breaking in our simulation matches the production of fragments where we see an increased ion yield in the experiment. This indicates that the dynamics of delocalized 3+ states formed after Auger decay are dominated by Coulomb repulsion. In a simplistic picture, this can be regarded as to spread of charge to the outer parts of the molecular ion, causing fragmentation of groups far away from the central sulfur bridge.

## Conclusions

We report a detailed analysis of X-ray induced fragmentation of electrosprayed [Cystine + H]^+^ molecular ions. Based on summed and partial ion-yield as a function of photon energy across the S L-edge, along with DFT and ROCIS simulations at resonant excitation energies and MD simulations at ionizing photon energies, we deduce the major fragmentation pathways. When the S 2p electrons are resonantly excited to the LUMO, which is mostly of S character, the disulfide bridge is broken, and the fragments undergo further dissociation into smaller fragments. Upon resonant excitation to LUMO+1/+2/+3, with the orbitals having C–C and S–C contributions, the S–C bonds are broken. The fragments formed in this way are also further broken into smaller fragments. On ionizing the S 2p orbital, the disulfide bridge remains intact and residual C_2_O_2_NH_4_^+^, COOH^+^, and C_2_NH_5_^+^ are formed. Thus, there is a photon energy-dependent fragmentation when the resonant excitation from the S 2p core level into the LUMO breaks the disulfide bridge, while excitation to higher unoccupied molecular orbitals and/or ionization leaves the bridge intact. The deduced fragmentation pathway provides an atomic picture of breaking a disulfide bridge using X-rays.

As disulfide bridges stabilize the protein structure, understanding the radiation-induced fragmentation pathway around the bridge is pivotal to deduce the protein structure imaging where the molecules are exposed to ionizing radiations. For example, in the developing single-particle imaging technique, heavy elements like sulfur can aid in determining the orientation of proteins, via the fragment distribution after the Coulomb explosion.^40^ To investigate to what extent resonant core-excitation affects the fragmentation process, it would be instructive to study longer carbon chains, where the electronic structure does not have time to relax fully before fragmentation. The S K-edge X-ray absorption near-edge spectra of solvated cystine have shown that excitation to the σ*SS orbital is unaffected by the presence of water, but that excitation to σ*S–C is sensitive to hydration.^41^ Moreover, K-edge absorption and fragmentation studies on gas-phase dimethyl disulfide show a preferential breaking of the S–S bond, even though fragments with intact S–S bonds could be observed at all excitation energies.^42^ Therefore, targeting the S K-edge of cystine can also result in different trajectories due to the significantly higher binding energy of the 1s electron, leading to several interesting Auger decay paths.

## Supporting Information Available

Unscaled partial and summed ion yield spectra and link to source files for geometry optimization and S L-edge calculation (PDF)
