# Supplementary material for: X-ray Induced Fragmentation of Protonated Cystine
Source: J Phys Chem A. 2022 Feb 25;126(9):1496–503. doi: 10.1021/acs.jpca.1c10158 (PMC8919253; doi:10.1021/acs.jpca.1c10158)
Supplement: Supplementary file 1 — jp1c10158_si_001.pdf [file jp1c10158_si_001.pdf]

Supporting Information: X-ray Induced Fragmentation of Protonated Cystine

Geethanjali Gopakumar,*,† Pamela H. W. Svensson,† Oscar Grånäs,† Barbara Brena,† Lucas Schwob,‡ Isaak Unger,† Clara-Magdalena Saak,¶ Martin Timm,§,‖ Christine Bülow,§,⊥ Markus Kubin,§ Vicente Zamudio-Bayer,§ J. Tobias Lau,§,⊥ Bernd von Issendorff,⊥ Abdul R. Abid,†,# Andreas Lindblad,† Emma Danielsson,† Ebba Koerfer,† Carl Caleman,†,@ Olle Björneholm,† and Rebecka Lindblad*,§,△,▽

†*Dept. Physics and Astronomy, Uppsala University, Box 516, SE-751 20 Uppsala, Sweden*

‡*Deutsches Elektronen-Synchrotron DESY, Notkestrasse 85, DE-22607 Hamburg, Germany*

¶*Dept. Physical Chemistry, University of Vienna, Währingerstraße 42, 1090 Vienna, Austria*

§*Abteilung für Hochempfindliche Röntgenspektroskopie, Helmholtz-Zentrum Berlin für Materialien und Energie, Albert-Einstein-Strasse 15, DE-12489 Berlin, Germany*

‖*Institut für Optik und Atomare Physik, Technische Universität Berlin, Hardenbergstrasse 36, DE-10623 Berlin, Germany*

⊥*Physikalisches Institut, Albert-Ludwigs-Universität Freiburg, Hermann-Herder-Strasse 3, DE-79104 Freiburg, Germany*

#*Nano and Molecular Systems Research Unit, University of Oulu, P. O. Box 3000, Finland*

@*Center for Free-Electron Laser Science, Deutsches Elektronen-Synchrotron DESY, Notkestrasse 85, DE-22607 Hamburg, Germany*

△*Dept. Physics, Lund University, Box 118, SE-22100 Lund, Sweden*

▽*Dept. Chemistry - Ångström Laboratory, Uppsala University, Box 538, SE-75121 Uppsala, Sweden*

E-mail: geethanjali.gopakumar@physics.uu.se; rebecka.lindblad@kemi.uu.se

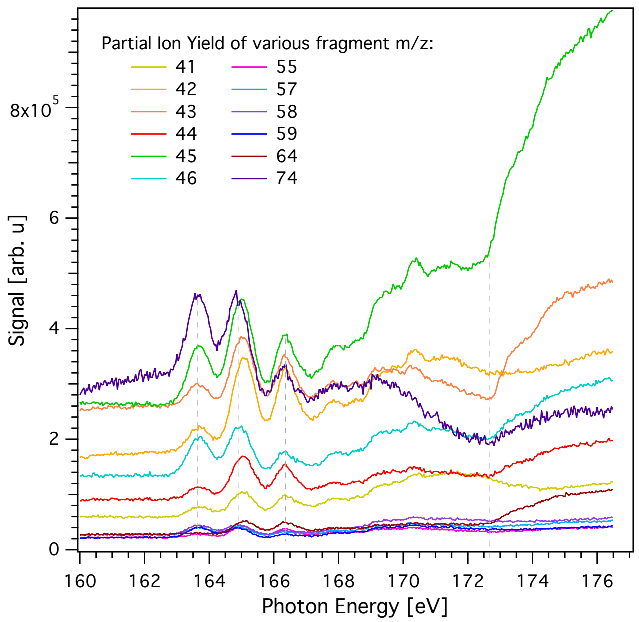

Figure SI.1: The partial ion yield of the different fragments (labelled by their m/z) without any scaling of the intensity.

The ion yield spectra of the different fragments presented in figure 2 in the main article are scaled for the purpose of clarity. Figure SI.1 shows the actual intensities of the ion yield spectra of the different fragments. The unscaled summed ion yield is shown separately in figure SI.2.

Source files for geometry optimization and S L-edge calculation can be found at `https://doi.org/10.5281/zenodo.5018348`.

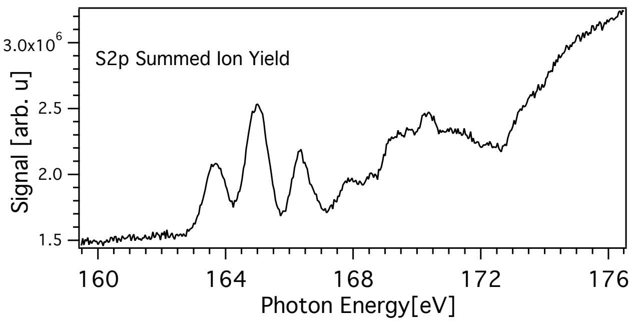

Figure SI.2: The summed ion yield of all fragments shown in Figure SI.1 without scaling of the intensity.
